# LncRNAs regulate the cytoskeleton and related Rho/ROCK signaling in cancer metastasis

**DOI:** 10.1186/s12943-018-0825-x

**Published:** 2018-04-04

**Authors:** Yanyan Tang, Yi He, Ping Zhang, Jinpeng Wang, Chunmei Fan, Liting Yang, Fang Xiong, Shanshan Zhang, Zhaojian Gong, Shaolin Nie, Qianjin Liao, Xiayu Li, Xiaoling Li, Yong Li, Guiyuan Li, Zhaoyang Zeng, Wei Xiong, Can Guo

**Affiliations:** 1 Department of Colorectal Surgery, Hunan Cancer Hospital and The Affiliated Cancer Hospital of Xiangya School of Medicine, Central South University, Changsha, Hunan China; 2 The Key Laboratory of Carcinogenesis and Cancer Invasion of the Chinese Ministry of Education, Cancer Research Institute, Central South University, Changsha, Hunan China; 3 The Key Laboratory of Carcinogenesis of the Chinese Ministry of Health, Xiangya Hospital, Central South University, Changsha, Hunan China; 4 Hunan Key Laboratory of Nonresolving Inflammation and Cancer, Disease Genome Research Center, The Third Xiangya Hospital, Central South University, Changsha, Hunan China; 5 Department of Cancer Biology, Lerner Research Institute, Cleveland Clinic, Cleveland, OH USA; 6 School of Electronics and Information Engineering, Hunan University of Science and Engineering, Yongzhou, Hunan China

**Keywords:** Long noncoding RNA, Cytoskeleton, Rho/ROCK signaling, Cancer metastasis

## Abstract

Some of the key steps in cancer metastasis are the migration and invasion of tumor cells; these processes require rearrangement of the cytoskeleton. Actin filaments, microtubules, and intermediate filaments involved in the formation of cytoskeletal structures, such as stress fibers and pseudopodia, promote the invasion and metastasis of tumor cells. Therefore, it is important to explore the mechanisms underlying cytoskeletal regulation. The ras homolog family (Rho) and Rho-associated coiled-coil containing protein serine/threonine kinase (ROCK) signaling pathway is involved in the regulation of the cytoskeleton. Moreover, long noncoding RNAs (lncRNAs) have essential roles in tumor migration and guide gene regulation during cancer progression. LncRNAs can regulate the cytoskeleton directly or may influence the cytoskeleton via Rho/ROCK signaling during tumor migration. In this review, we focus on the regulatory association between lncRNAs and the cytoskeleton and discuss the pathways and mechanisms involved in the regulation of cancer metastasis.

## Background

Cell migration requires cytoskeletal reorganization, which plays a critical role in cancer metastasis [1, 2]. In particular, some classical signaling pathways are involved during cytoskeletal reorganization, such as the ras homolog family (Rho) and Rho-associated coiled-coil protein kinase (ROCK) signaling pathway [3, 4].

Approximately 75% of human genomic DNA can be transcribed into RNA, but only 2–3% of the genes encode a protein; therefore, in the entire transcriptome, the proportion of noncoding RNAs is far higher than the proportion of protein-coding mRNAs [5]. Among the former, lncRNA is a transcript longer than 200 nucleotides and cannot code for a protein; it can play an important role in the process of tumor metastasis through multiple mechanisms [6]. The abnormal expression of lncRNAs is related to the poor prognosis of patients with cancer. Studies have revealed that the lncRNA AFAP1-AS1 promotes the invasion and migration of nasopharyngeal carcinoma (NPC) cells by stimulating stress fiber formation [7]. The lncRNA Pvt1 oncogene (PVT1) is related to poor prognosis by inhibiting apoptosis in colorectal cancers [8]. PVT1 is an indicator of poor prognosis for gastric cancer and promotes cell proliferation by regulating the epigenetic expression of p15 and p16 [9]. Depletion of the lncRNA urothelial cancer associated 1 (UCA1) induces radiosensitivity and decreases proliferative capacity [10]. Thus, lncRNAs can promote tumor cell invasion, migration, proliferation, and radiosensitivity and inhibit tumor cell apoptosis, thereby accelerating tumor progression, predisposing cancer patients to distant metastasis, and resulting in poor prognosis.

LncRNAs can directly interact with cytoskeletal proteins to change the three-dimensional structure of cells and can regulate the cytoskeleton through the Rho/ROCK signaling pathway. Therefore, the interactions among lncRNAs, Rho/ROCK signaling, and the cytoskeleton underlie the ability of a cell to become motile, eventually leading to tumor migration [7, 11]. This review describes the current knowledge about the mechanisms underlying cytoskeleton reprogramming, followed by discussion of the roles of lncRNAs. The interaction between lncRNAs and cell migration suggests new therapeutic targets in cancer metastasis.

## Cytoskeletal reorganization and cancer cell movement

The cytoskeleton refers to the structure of the protein fiber network in eukaryotic cells. It plays an important role in the maintenance of cell shape, cell movement, transportation of substances in cells, and cytokinesis. Eukaryotic cells contain three main types of cytoskeletal filaments: microfilaments, microtubules, and intermediate filaments [12].

Microfilaments are also called actin filaments. They are approximately 7 nm in diameter and are composed of two strands of spiral fibers, which are formed by actin polymerization [13]. The basic unit of the microfilament is globular actin (also known as G-actin). Actin monomers, one after another, link to form an actin chain, and two actin chains twist around each other to form a strand of microfilament [14]. This actin polymer is filamentous actin (F-actin). The main functions of microfilaments are the formation of stress fibers, cell movement, and cytokinesis [15].

The structure and function of microfilaments is regulated by a variety of proteins. These proteins are associated with the microfilament and are known as microfilament-associated proteins; some examples include capping proteins, Arp2/3 complex, cofilin, etc. [16, 17].

The capping protein can selectively block one end of filamentous F-actin, resulting in the shortening or extension of the microfilament, which is vital for the high motility of cancer cells [18]. Research has shown that the capping protein scinderin (SCIN) regulates actin and then participates in the migration of tumor cells. In addition, the silencing of SCIN in vitro and in vivo significantly inhibits the formation of filopodia and reduces the migratory ability of gastric cancer cells [19]. The Arp2/3 complex is an actin-related protein and can nucleate the microfilament (nucleation) [20]. The entire complex can be bound to the microfilament to allow new microfilaments to be generated. In tumor cells, the function of the Arp2/3 complex is related to the movement of the lamellipodia [21]. Cofilin is a family of actin-binding proteins that disassemble microfilaments [22]. The depolymerization of cofilin can change the adhesion between cells and the extracellular matrix and ultimately promote cell migration [23, 24]. Fascin is a type of actin-bundling protein [25]. The primary functions of fascin are related to adhesion and the formation of filopodia in the movement of cancer cells [26].

Microtubules have a diameter of 25 nm [27]. They are composed of α and β tubulin subunits that form tubulin dimers [28]. Microtubules function by resisting compression and bending to maintaining cell morphology [29]. During cell migration, microtubules, together with the attached dynein, may release a signal to promote focal adhesion depolymerization [30].

The diameter of an intermediate filament is 10 nm, which is the size between microtubules and microfilaments. This structure is the most stable cytoskeletal component and plays a major role in supporting the cell [31]. Microtubules and microfilaments are assembled by spherical proteins, and intermediate fibers are assembled by long, rod-shaped proteins [32]. The intermediate filament proteins can be divided into six types based on similarities in amino acid sequence and protein structure: acidic and basic keratins, vimentin, neurofilaments, nuclear lamin, and nestin. Vimentin is the most widely distributed of all intermediate filament proteins [33–35]. Vimentin regulates cell adhesion molecules and other components and is involved in tumor cell adhesion, the epithelial–mesenchymal transition (EMT), migration, and invasion [36–38].

When invading the dense extracellular matrix, cancer cells need to have a high degree of deformability. The network structure of fibers that is composed of microfilaments and various microfilament binding proteins under the plasma membrane is called the cell cortex [39]. The cell cortex can push the cell membrane to form protrusions, including lamellipodia, filopodia, and invadopodia [40].

Lamellipodia are at the front end of a migrating cell and contain actin filament branching structures that form an actomyosin branch, which is more mature, and then extend to form lamellar protrusions. Lamellipodia usually help the cell move forward and can also drive random or continuous cell migration that is associated with the migratory phenotype of tumor cells [41]. Lamellipodia generate the driving force for cell migration [42].

Filopodia are small fingerlike cell protrusions. They contain an F-actin parallel fascicular arrangement. Filopodia are formed by actin polymerization, extending to the front end of the cell, and are especially prominent in migrating cells [43]. The contractility of filopodia is weaker than that of lamellipodia. Nonetheless, in some highly invasive tumor cells, lamellipodia are not formed, but a large number of filopodia can be observed [44, 45]. Therefore, filopodia may be associated with the invasive phenotype of tumor cells.

The invadopodia is rich in actin microfilaments and adhesion proteins, such as integrin, focal adhesion kinase (FAK), and vinculin, and forms a ring around the actin bundles [13]. The formation of invadopodia releases a variety of matrix metalloproteinases (MMPs) and degrades the extracellular matrix (ECM) to promote cell invasion [46]. ECM degradation mediated by invadopodia can also generate cell traction, which helps tumor cells to invade new sites [47].

**Fig. 1:**
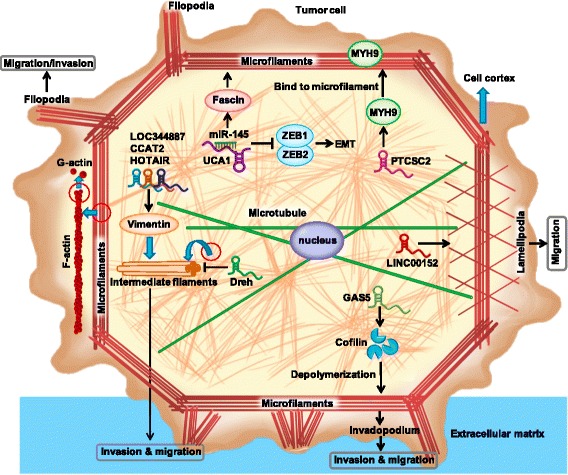
LncRNAs regulate the cytoskeleton and related proteins in cancer. LncRNAs can directly regulate the cytoskeleton and related proteins in various cancers. The lncRNA Dreh can inhibit the expression of intermediate filaments by binding to them and can prevent metastasis by changing the cytoskeletal structure and morphology of cancer cells. The lncRNA LINC00152 is involved in cytoskeletal remodeling and affects F-actin reorganization. LOC344887, CCAT2, and HOTAIR regulate vimentin. PTCSC2 regulates actin filaments via binding to MYH9

Interestingly, fast moving cells mainly form lamellipodia, while nearly immobile cells have lamellipodia and filopodia. Lamellipodia, filopodia, and invadopodia can remodel the cytoskeleton by changing microfilaments, microtubules, and intermediate filaments. In the process of tumor cell migration, cytoskeletal reorganization is regulated by non-coding RNA and signaling pathways [48, 49] (Fig. 1).

## LncRNAs regulate the cytoskeleton in cancer

lncRNAs can affect the migration of tumor cells by regulating the cytoskeleton or related proteins [50–52]. The most direct evidence comes from the function of a lncRNA called “downregulated in hepatocellular carcinoma” (Dreh). The expression of Dreh is lower in cancer tissues than that in normal tissues. Patients with high expression of Dreh show a low recurrence rate and long survival time. Dreh can inhibit cell migration, and further research showed that Dreh binds to and inhibits intermediate filaments and prevents cancer cell metastasis by changing the cytoskeletal structure and cell morphology [53].

Another cytoskeleton-related lncRNA is LINC00152, which also known as cytoskeleton regulator RNA. The expression of LINC00152 is upregulated in tissue samples from various cancers. For example, Chen et al. showed that LINC00152 expression is increased in 60 human lung adenocarcinoma tissue samples relative to paired normal tissues [54]. Müller et al. reported the upregulation of LINC00152 in pancreatic cancer tissue [55]. LINC00152 is associated with a poor prognosis of tongue carcinoma with invasion and metastasis and is overexpressed in a variety of tumors, such as breast cancer, lung cancer, gastric cancer, liver cancer, gallbladder cancer, and colorectal cancer. Therefore, LINC00152 can serve as a new tumor marker [54, 56–61]. In the breast cancer cell line MDA-MB-231, LINC00152 regulates target genes involved in cytoskeletal remodeling, including tubulin tyrosine ligase, Rho guanosine triphosphatase (GTPase) Rhobtb3, and plakophilin 4 [60]. An Ingenuity Pathway Analysis revealed that some of the target genes of LINC00152 are closely related to the cell spreading pathway, including actin polymerization–driven processes, the Rho family of GTPase promoters, and the mTORC2 complex [62, 63]. Therefore, in experiments that knocked down LINC00152 expression using a locked nucleic acid or that used fluorescently labeled F-actin, cells treated with LINC00152 are smaller, rounder, show actin reorganization, a reduction in stress fibers, and the appearance of thick actin fibers in the cortex compared with the control group [60]. In addition, according to the literature, LINC00152 may affect F-actin reorganization by regulating the expression of Golgi phosphoprotein 3 (GOLPH3) [64].

Vimentin is responsible for maintaining the integrity of the cytoskeleton and cell shape [65]. Many lncRNAs have been shown to affect vimentin, including HOX transcript antisense RNA (HOTAIR), LOC344887, and colon cancer associated transcript 2 (CCAT2). HOTAIR is relevant to small cell lung cancer invasiveness by suppressing cell adhesion–related genes such as astrotactin 1 (ASTN1) and protocadherin alpha 1 (PCDHA1) [66]. In cervical carcinoma, HOTAIR promotes the migration and invasiveness of HeLa cells by regulating the expression and organization of vimentin [51]. The inhibition of HOTAIR significantly promotes the collapse of the vimentin intermediate-filament network to lead to a decrease in cell migration and invasion [51].

Vimentin is a marker of EMT that appears during cancer metastasis. LOC344887 increases the migration and invasion of gallbladder cancer cells. In particular, LOC344887 leads to EMT by increasing the protein expression of vimentin [67]. Similarly, the lncRNA CCAT2 promotes hepatocellular cancer metastasis by positively regulating vimentin and inducing EMT [68]. Vimentin intermediate filaments are strongly involved in cell shape, focal adhesion, and motility by altering these characteristics during EMT [69].

The lncRNA papillary thyroid cancer susceptibility candidate 2 (PTCSC2) has one unspliced isoform and several spliced isoforms, all of which show thyroid-specific expression. Myosin-9 (MYH9) interacts with the lncRNA PTCSC2 [70]. Further studies have shown that MYH9 binds to the FOXE1 promoter region, PTCSC2, to regulate FOXE1 promoter activity. MYH9 participates in the generation of cell polarity, cell migration, cell–cell adhesion processes, and cytoskeleton maintenance by binding to actin filaments [71].

The expression of the lncRNA growth arrest–specific 5 (GAS5) is downregulated in glioma tissues. GAS5 inhibits the migration and invasion of U87 and U251 human glioma cell lines [72]. Mechanistically, overexpression of GAS5 increases the expression of plexin C1, which encodes a member of the plexin family. Plexins are transmembrane receptors for semaphorins, which regulate cell motility and migration by downregulating miR-222 [72]. Furthermore, plexin C1 targets cofilin by inducing cofilin inactivation rather than by decreasing the cofilin amount. Cofilin stimulates microfilament disassembly to promote cell mobility during tumor migration and invasion [72, 73]. This finding may indirectly explain why GAS5 inhibits the migration and invasion of glioma cells by reorganizing the cytoskeleton. Nevertheless, the mechanism needs further study.

The lncRNA UCA1 is upregulated in bladder cancer and induces EMT, migration, and invasion of bladder cancer cells. Mechanistically, UCA1 regulates bladder cancer cell migration and invasion by miR-145 and its target genes actin-bundling protein fascin and zinc finger E-box binding homeobox 1 and 2 (ZEB1/2) [74]. UCA1 is a direct target of hsa-miR-145 by interacting with the miR-145 binding site at exons 2 and 3 of UCA1. UCA1 mediates bladder cancer migration and invasion through the miR-145-ZEB1/2-fascin pathway [74]. Fascin localizes along the entire length of all filopodia. RNA interference of fascin reduced the number of filopodia, and the remaining filopodia had abnormal morphology and loosely bundled actin organization [75, 76].

Thus, different lncRNAs can affect the migration of tumor cells via affecting various components of the cytoskeleton and associated proteins (Fig. 1 and Table 1).

**Table 1:** Summary of lncRNAs regulate cytoskeletons in cancer

LncRNAs	Cytoskeletons and associated protein	Functions	Refs
Dreh	Intermediate filament	Prevent cancer cell migration through changing the cytoskeleton structure and cell morphology	[34]
LINC00152	F-actin	LINC00152 affects F-actin by regulating the expression of GOLPH3	[41, 45]
LOC344887, CCAT2, HOTAIR	Vimentin	LOC344887, CCAT2 and HOTAIR regulate the expression of vimentin	[32, 55, 57]
PTCSC2	MYH9	PTCSC2 binds MYH9 to regulate cytoskeleton	[59]
GAS5	cofilin	GAS5 suppressed glioma cell growth, migration and invasion by targeting miR-222. miR-222 induced cofilin dephosphorylation by silencing PLXNC1 gene	[61]
UCA1	FSCN1	UCA1 promotes bladder cancer cell migration and invasion by the hsa-miR-145–ZEB1/2– FSCN1 pathway	[66]

## Rho/ROCK signaling in cytoskeletal reorganization

Many receptor proteins activated in the plasma membrane can initiate cytoskeletal reorganization; the signals are all mediated by the RhoGTP family and the downstream effector ROCK, which compose the Rho/ROCK signaling pathways [77, 78]. The most studied Rho GTPases can be subdivided into three classes: Rho (RhoA, RhoB, and RhoC), Rac (Rac1, Rac2, and Rac3), and cell division cycle 42 (Cdc42). Other less studied GTPases include RhoD and RhoE [79–81].

Rho aggregates actin and myosin to form stress fibers and focal adhesion complex assembly [82, 83]. RhoA is present at the cell membrane when it is active [84]. RhoA regulates the generation of actomyosin bundles, stress fibers, focal adhesions, and lamellipodia [85]. RhoB is found in endosomes and at the plasma membrane. The role of RhoB in cancer progression remains unknown, and it responds to specific signals in the tumor microenvironment [84]. RhoC modulates phagosome formation by actin cytoskeletal remodeling via mDia1 [86].

Rac primarily promotes the formation of lamellipodia and invadopodia. Rac1 localizes mainly to the plasma membrane and drives the formation of lamellipodia and invadopodia [87]. Rac2 is critical for cell adhesion to intercellular adhesion molecule-1 (ICAM-1) and for immunological synapse formation [88]. Rac3 is critical for integrating the adhesion of invadopodia to the extracellular matrix (ECM) to allow invadopodia to degrade the ECM [89].

Cdc42 promotes the formation of filopodia and induces cell migration and metastasis [90]. Other less studied GTPases, such as RhoE, can bind to ROCK1 and inhibit the activity of ROCK1 [91, 92]. Phosphoinositide dependent kinase 1 activates ROCK1 by opposing the inhibition of RhoE and then promotes cell motility [92].

The ROCK family includes two members, ROCK1 and ROCK2 [93], which are encoded by two different genes [94, 95]. ROCK1 plays a key role in the formation of stress fibers, and this isoform is mainly responsible for rigidity-dependent invadopodia activity through actomyosin contractility [96, 97]. ROCK2 is important for phagocytosis, cell contraction and stabilizing the cytoskeleton [78, 97, 98].

Rho/ROCK signals can regulate the development and balance the formation of lamellipodia, filopodia and invadopodia, and these signals promote the degradation of the extracellular matrix [99].

Rac1 promotes actin polymerization during lamellipodium formation through the WAVE complex and subsequent activation of the Arp2/3 complex [100].

Cdc42 activates the formin protein mDia2 to regulate actin nucleation and elongation of microfilaments [101, 102]. Cdc42 can also activate N-WASP to stimulate the Arp2/3 to induce actin polymerization. The straight parallel alignment of microfilaments form filopodia [99].

RhoA and RacC play important roles during the formation of invadopodia. RhoA activates mDia2 and further induces the formation of linear actin bundles to result in the elongation of invadopodia [103]. ET-1 triggers increased binding to ETAR and promotes the formation of the β-arrestin/PDZ-RhoGEF signaling complex, which activates ROCK/LIMK/cofilin through RhoC activity and generates actin remodeling and invadopodia formation [104].

**Fig. 2:**
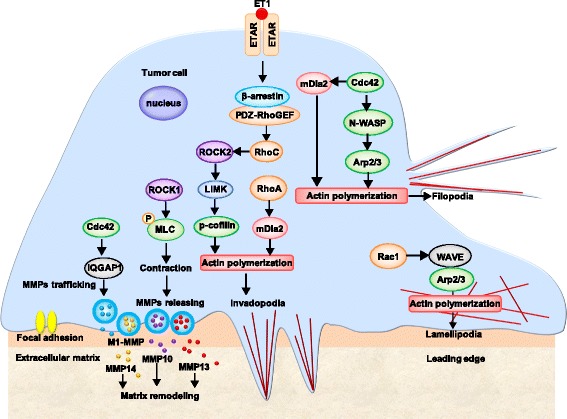
Rho/ROCK signaling in cytoskeleton reorganization. Rho/ROCK signaling regulates the formation of lamellipodia, filopodia and invadopodia and the release of MMPs

In addition to regulating the formation of invadopodia, Cdc42 plays an important role in the trafficking of MMPs to the invadopodia. For example, Cdc42 induced IQGAP1 binding at invadopodia to traffic MT1-MMP and MMP14 by vesicles [105, 106]. ROCK expression increases during pancreatic cancer progression, and ROCK consequently increases the phosphorylation of MLC2. pMLC2 causes actomyosin contraction and then induces the release of the stromelysin MMP10 and the collagenase MMP13. Eventually, MMPs promote extracellular matrix remodeling to enable invasive growth [94] (Fig. 2).

## LncRNAs regulate Rho/ROCK signaling during tumor migration

LncRNAs can regulate cancer cell migration by targeting Rho/ROCK signaling and include metastasis-associated lung adenocarcinoma transcript 1 (MALAT1), actin filament associated protein 1 antisense RNA1 (AFAP1-AS1), and maternally expressed 3 (MEG3) [7, 107, 108]. The lncRNA MALAT1 reduces the protein expression levels of RhoA, ROCK1, and ROCK2, indicating that MALAT1 may promote osteosarcoma cell migration by the RhoA/ROCK pathway; then, MALAT1 increases the number of actin stress fibers in osteosarcoma cells [107]. Another study suggested that MALALT1, miR-1, and cdc42 are competitive endogenous RNAs (ceRNAs) in breast cancer cells [108]. MALAT1 can bind and inhibit miR-1; miR-1 can bind to the 3’UTR of cdc42 and decrease the expression of cdc42 to induce the migration and invasion of breast cancer cells [108].

Our research group has found that the lncRNA AFAP1-AS1 leads to the loss of stress fiber formation in nasopharyngeal carcinoma by influencing the expression of RhoA/Rac2 signaling and F-actin polymerization [7]. Zhang et al. also found that increased expression of AFAP1-AS1 significantly correlates with pathological staging and lymph-vascular space invasion in patients with hepatocellular carcinoma via inhibition of RhoA/Rac2 signaling. Overall, AFAP1-AS1 may promote NPC and hepatocellular carcinoma metastases through RhoA/Rac2 signaling [109].

Wang et al. demonstrated that downregulated lncRNA MEG3 is associated with lymph node metastasis in primary thyroid cancer. Mechanistically, MEG3 suppresses the expression of Rac1 through a specific site in the 3′UTR [110]. Vinculin is another motility-associated protein that is synthesized in migrating cells, and vinculin-deficient cells extend unstable lamellipodia and filopodia. The lncRNA XLOC010623 activates the TIAM1/Rac1 and RhoA/ROCK2 signaling pathways, increases the expression of vinculin and causes the migration of adipose tissue–derived stem cells [111]. The lncRNA SchLAH physically inhibits the migration of HCC cells through RhoA and Rac1 [112]. The regulatory mechanism of the lncRNAs ABHD11-AS1 and TDRG1 are similar; they both induce the expression of RhoC and MMP during tumor progression [113, 114].

**Fig. 3:**
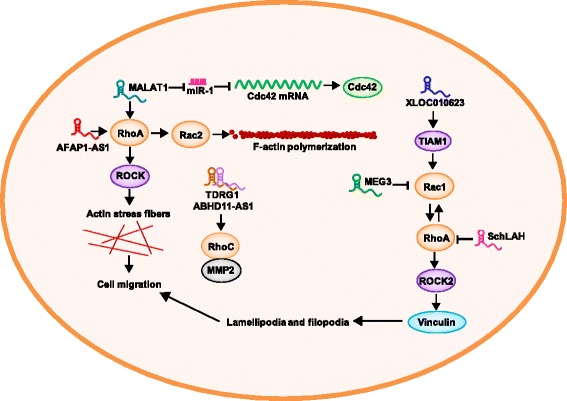
LncRNAs regulate Rho/ROCK signaling in cancer metastasis. MALAT1 facilitates the metastasis of osteosarcoma though the RhoA/ROCK pathway and can act as ceRNA to inhibit miR-1 by targeting Cdc42, thereby inducing breast cancer cell metastasis. XLOC010623 activates the TIAM1/Rac1 and RhoA/ROCK2 signaling pathways and then increases the expression of vinculin, leading to the migration of adipose tissue–derived stem cells. SchLAH physically inhibits the migration of HCC cells through RhoA and Rac1. MEG3 downregulates Rac1 expression and is linked to primary thyroid cancer with lymph node metastasis. AFAP1-AS1 might promote NPC and HCC metastasis through RhoA/Rac2 signaling. TDRG1 induces RhoC expression and tumor progression in EOC. ABHD11-AS1 can directly bind to RhoC and upregulate the expression of its downstream molecule MMP2, thus increasing the metastatic ability of EOC

LncRNAs are involved in cancer metastasis mainly through reorganizing the cytoskeletal structure and by regulating the expression of molecules in the RhoA/ROCK pathway to result in an increased number of actin cytoskeleton fibers, stress fiber formation, formation of lamellipodia and filopodia, tumor cell adhesion, and angiogenesis. The regulatory mechanism includes ceRNA and direct binding to RAC1 and other molecules in the Rho/ROCK pathway. Understanding these relationships may provide insights into human lncRNA regulation, cytoskeletal structure, cell migration, and cancer metastasis. We present various lncRNAs in Fig. 3 and Table 2 and describe many lncRNAs that regulate tumor metastasis through cytoskeletal remodeling via the Rho/ROCK pathway. lncRNAs may be a promising target for future cancer therapy.

**Table 2:** Summary of lncRNAs regulate the Rho/ROCK signaling in cancer

LncRNAs	Rho/Rho-associated protein	Functions	Refs
MALAT1	RhoA, ROCK1 and ROCK2	MALAT1 facilitates the metastasis of osteosarcoma though RhoA/ROCK pathway	[103]
MALAT1	Cdc42	MALAT1 plays a role of Cdc42 ceRNA and induces migration by blocking miR-1 in breast cancer cells	[104]
AFAP1-AS1	RhoA/Rac2	AFAP1-AS1 might promote tumor metastasis through regulation of tumor cell adhesion and mobility via RhoA/Rac2 signaling	[7]
MEG3	Rac1	MEG3 is a novel suppressor of migration and metastasis by targeting Rac1 gene	[107]
XLOC 010623	Rac1, RhoA and ROCK2	Tetrahedral DNA nanostructures (TDNs) suppressed the transcription of XLOC010623, and activated the TIAM1/Rac1 and RhoA/ROCK2 to promote cell migration	[108]
SchLAH	RhoA and Rac1	SchLAH suppressed migration of HCC cells through downregulating RhoA and Rac1	[110]
TDRG1	RhoC	TDRG1 induced RhoC expression in epithelial ovarian carcinoma	[111]
ABHD11-AS1	RhoC	ABD11-AS1 can bind to RhoC directly in epithelial ovarian carcinoma	[112]

## Conclusions

From a physics viewpoint, the development of a tumor is a biological process driven by mechanics, which is regulated by the biochemical signaling pathways of tumor cells. For example, changes in cellular mechanical properties can activate signal transduction pathways. In this review, we associate the physical movement of the cell with Rho/ROCK signaling and discuss the regulatory involvement of lncRNAs, which are significant for future research.

Many studies have shown that Rho/ROCK-mediated cytoskeletal regulation plays a key part in cancer metastasis. External factors stimulate the transition of normal cells to tumor cells. The activity of the intracellular Rho signal increases, the arrangement of cytoskeleton fibers changes, and then cell morphology is affected. In contrast, in the Rho/ROCK pathway, lncRNA-regulated specific molecules are less frequent, and we do not understand their specific mechanisms of action. Therefore, the mechanism of Rho/ROCK regulation by lncRNA and their relationship to metastasis remain to be studied.

Targeting Rho/ROCK signaling-associated lncRNAs could be useful for inhibiting the migration of cancer cells and may be a new target for the treatment of cancer metastasis. Some questions need to be addressed. For example, do lncRNAs regulate other signaling pathways that are associated with the cytoskeleton? How can the cytoskeleton be targeted via lncRNAs for clinical cancer treatment? With the development of lncRNA research and technologies for measuring cell movements, the relationship between lncRNAs and cell migration and invasion in tumor metastasis can be uncovered.

## Abbreviations

AFAP1-AS1: actin lament associated protein 1 antisense RNA1
ASTN1: astrotactin 1
CCAT2: colon cancer associated transcript 2
CDC42: cell division cycle 42
ceRNA: competitive endogenous messenger RNA
Dreh: downregulated in hepatocellular carcinoma
EMT: epithelial mesenchymal transition
F-actin: filamentous actin
FSCN1: fascin actin-bundling protein 1
GAS5: Growth Arrest­Specific 5
GTPase: Rho guanosine triphosphatase
HOTAIR: Hox transcript antisense intergenic RNA
LIMK: LIM kinase
LncRNAs: long noncoding RNAs
MALAT1: metastasis-associated lung adenocarcinoma transcript 1
MEG3: maternally expressed 3
MLC: myosin light chain
MYH9: Myosin-9
MYPT1: myosin phosphatase 1
NPC: nasopharyngeal carcinoma
PAK: p21 activated kinase
PCDHA1: protocadherin alpha 1
PTCSC2: papillary thyroid cancer susceptibility candidate 2
PVT1: Pvt1 oncogene
RhoA: Ras homolog family member A
ROCK: Rho-associated coiled-coil containing protein serine/threonine kinases
TDRG1: testis development–related gene 1
UCA1: urothelial cancer- associated 1
